# Molecular characterization and risk analysis of *Giardia duodenalis* assemblages in corticosteroid-treated and non-treated patients in Ismailia, Arab Republic of Egypt

**DOI:** 10.1186/s13099-024-00668-y

**Published:** 2024-12-13

**Authors:** Shahira Abdelaziz Ali Ahmed, Amira Bakr Mokhtar, Samar Farag Mohamed, Marwa Ibrahim Saad El-Din, Catherine O’Dowd Phanis, Stefani Kazamia, Chad Schou, Paweł Gładysz, Anna Lass, Annalisa Quattrocchi, Panagiotis Karanis, Samer Eid Mohamed Gad

**Affiliations:** 1 Department of Parasitology, Faculty of Medicine, Suez Canal University, Ismailia, 41522 Egypt; 2 Department of Family Medicine, Faculty of Medicine, Suez Canal University, Ismailia, 41522 Egypt; 3 Invertebrates- Zoology Department, Faculty of Science, Suez Canal University, Ismailia, 41522 Egypt; 4 Department of Basic and Clinical Sciences, University of Nicosia Medical School, 24005, CY-1700 Nicosia, Cyprus; 5 Department of Tropical Medicine and Parasitology, Institute of Maritime and Tropical Medicine in Gdynia, Gdynia, Poland; 6 Department of Primary Care and Population Health, University of Nicosia Medical School, 24005, CY-1700 Nicosia, Cyprus; 7 Medical Faculty and University Hospital, University of Cologne, Cologne, Germany

**Keywords:** *Giardia duodenalis*, Assemblage/sub-assemblage, Corticosteroid therapy, Case–control, Multi-locus sequence typing, Egypt

## Abstract

**Background:**

*Giardia duodenalis* (*G. duodenalis*) is an intestinal protozoan parasite of human and animal hosts. The present study investigated and compared the assemblages of *G. duodenalis*-infected faecal samples in patients on corticosteroid therapy (POCT) and control patients-not on corticosteroid therapy (CONT) and differentiated its assemblages and/or sub-assemblages’ relationship with associated risk factors.

**Methods:**

Utilizing multi-locus sequence typing (MLST) with three loci targeted—triosephosphate isomerase (*tpi*), ꞵ-giardin (*bg*), and glutamate dehydrogenase (*gdh*)—*G. duodenalis* isolated from POCT and CONT were analyzed. Risk factors linked with *Giardia* infection and its assemblages were investigated.

**Results:**

In total, 52 *G. duodenalis*-infected patients were enrolled: 21 POCT and 31 CONT. The mean age was 12.3 years, the majority were male (59.6%), and 73.1% lived in rural areas. The POCT group was 36 times more likely than the CONT group to have a concurrent parasitic infection. About 73% (38/52) of *Giardia* samples were genotyped and/or sub-genotyped in at least one of the three loci. MLST identified sixteen isolates (42.0%) as assemblage B, ten isolates (26.3%) as assemblage A, and twelve isolates (31.6%) as a mixed infection of A + B and B + E. Most individuals of the POCT group were infected with *G. duodenalis* assemblage A while most of the CONT group were infected with assemblage B. Sub-assemblage AII was identified by phylogenetic analysis in the isolates of both groups under investigation.

**Conclusion:**

This research advances giardiasis epidemiology in Arab Republic of Egypt (ARE) and reflects how corticosteroid-treated patients differ from those non-treated in *Giardia* assemblage pattern and their susceptibility to concomitant infection. Overall, *Giardia* assemblage patterns in this research populations reflect anthroponotic and zoonotic transmission, emphasizing the importance of public health policy and giardiasis prevention of illness transmission, particularly among those on corticosteroid therapy in ARE.

**Supplementary Information:**

The online version contains supplementary material available at 10.1186/s13099-024-00668-y.

## Introduction

As the most common protozoan pathogen globally, *Giardia duodenalis* (*G. duodenalis*) is a unicellular intestinal flagellate that is frequently found in the digestive tracts of human and animals [1]. Infection with *G. duodenalis* occurs through the ingestion of cysts present in contaminated water and food or by direct person-person or animal-person contact [2]. The cyst form exhibits resistance to environmental conditions and can persist for prolonged durations in cool, moist environments, promoting the spread of giardiasis [2]. Giardiasis was included in the Neglected Disease Initiative target by the World Health Organization (WHO) in September 2004 because of its effects on the health of expectant mothers and children as well as its connection to poverty [3]. *Giardia* is frequently cited as a causative agent in global waterborne outbreaks accounting for 448 epidemics over the last five decades [4–7]*.* In developed countries, the estimated prevalence of *G. duodenalis* can range between 2 to 5%. However, resource-poor nations exhibit a significantly higher prevalence of 20–30% due to substandard sanitation, hygiene, and water supplies [8]. *G. duodenalis* infects 300 million individuals in Africa, Asia, and Latin America, with the majority of those afflicted being children living in low-income settings [9].

The severity and progression of a *G. duodenalis* infection usually depends on the health status and age of the individual, the number of cysts introduced to the host, and the virulence of the parasite's variants [10]. In immunocompetent humans, infection with *G*. *duodenalis* is primarily asymptomatic; however, a variety of gastrointestinal manifestations have been documented from patients, including bloating, diarrhoea, flatulence, fatigue, nausea, steatorrhea and weight loss [11]. Although *Giardia* infection is not life-threatening, it can lead to severe infection in immunocompromised individuals. Refractory giardiasis, chronic diarrhoea over six months, sever infection and higher parasitic load have been reported in immunocompromised patients like hypogammaglobulinemia and nephrotic syndrome, cancer, and renal transplant [12–15]. Furthermore, giardiasis can cause a wide range of extra-intestinal symptoms, such as hypokalemic myopathy, ocular diseases, arthritis, allergies, decreased cognitive function and failure to thrive in children [16].

The long-term consequences mainly affect the elderly, newborns, young children, travelers, institutionalized individuals, and individuals with weakened immune systems, such as those receiving corticosteroid therapy or those with acquired immunodeficiency syndrome (AIDS) [16, 17]. Regardless of immune status, giardiasis has a large global prevalence [18, 19]. However, many of the documented human adult cases have been self-limiting illnesses [10, 20].

*G. duodenalis* has been characterized as a multispecies complex using iso-enzymatic and nucleic acid polymorphism investigations. There are eight *Giardia* species that are recognized on distinct genetic characteristics [21]. It consists of eight genetic assemblages (A–H) that are directly linked to a certain host group or human or animal [22]; assemblages C and D are primarily found in dogs; assemblage E primarily affects hoofed mammals; assemblages F, G, and H are exclusive to cats, rodents, and pinnipeds; assemblages A and B are commonly diagnosed in humans and other animal species [21]. Comparisons from multi-locus genotyping (MLG) techniques have also identified putative sub-assemblages within assemblage A (AI–III) and assemblage B (BIII and BIV) [23].

The possibility to distinguish between the genetic diversity of a population dynamics within a specific *G. duodenalis* assemblage has improved with the use of multiple genetic marker analysis [24]. Single-locus genotyping data and genetic information are insufficiently sensitive to identify mixed infections and do not offer enough clues about the potential source of zoonotic transmission. Multi-locus sequence typing, on the other hand, allows for the possibility to indicate the zoonotic source of the human pathogenic assemblages A and B and offers improved confirmation and identification of mixed infections with distinct assemblages in the same specimen [25]. The β-giardin gene (*bg*), glutamate dehydrogenase gene (*gdh*), triosephosphate isomerase gene (*tpi*), and small subunit ribosomal RNA are among the loci that are frequently applied to detect numerous variations of *G. duodenalis* in different host species [24].

Glucocorticoids are commonly administered to interfere with the immune system due to their powerful anti-inflammatory and immunosuppressive properties. This class of medication significantly affects cell redistribution and maturation in lymphoid organs, immune response assembly, and polymorphonuclear cell adhesion and migration. Chronic users of anti-inflammatory medications and immunosuppressants may show challenges in resolving giardiasis [26]. However, *Giardia*-infected-immunosuppressed individuals might remain asymptomatic for long periods of time [26, 27].

In the Arab Republic of Egypt (ARE), *Giardia* infections have been frequently reported in the immunocompetent and immunocompromised individuals [28–40]. A group of immunocompromised Egyptian patients with diabetes mellitus, chronic renal failure, and cancer were reported to have *G. duodenalis* infections [33]. In a different study, the prevalence of *Giardia* infection was greater in diabetic patients (22%) compared to the control group (5%) [38]. Among immunocompromised patients, those receiving steroid therapy were found to be the most affected by *Giardia* infection [39]. Studies that utilized molecular biology techniques have identified assemblages A, B, C, and occasionally E in the Egyptian population in which restriction fragment length polymorphism or sequencing approaches were applied to a specific single genetic locus to identify these assemblages [36, 37, 40–48].

Additional information is needed about the molecular assemblages and genetic diversity of giardiasis infection in patients on corticosteroid therapy compared to control subjects. It is beneficial to ascertain any differences between the *G. duodenalis* infection status of these groups that might have an impact on future treatment protocols. This study aims to examine and identify the *G. duodenalis* assemblages in patients receiving corticosteroid therapy (cases) versus control individuals (controls), and to explore potential links between clinical symptoms and the identified assemblages or sub-assemblages.

## Methodology

### Study area and samples collection.

A case–control study was performed in Ismailia governorate, ARE. The study was carried out on patients attending the Family Practice Center (FPC) outpatient clinic affiliated to the Suez Canal University (SCU).

Written consent was a requirement for approval. In certain individuals who have a low literacy rate, verbal consent was granted. Witnessed by an FPC-clinic physician or chief nurse, parents’ or legal guardians’ consent for minors was obtained. Strict confidentiality and privacy were ensured. Anonymity was maintained throughout the analysis of all samples.

Following the history, the participants were provided a sterile, labelled plastic container with a collection stick for transferring faecal samples. Patients received explicit verbal instructions regarding the stool sample collection process. At least one faecal sample was obtained from each participant.

Faecal samples were collected at SCU-FPC and sent to the SCU-Parasitology Laboratory. While samples from individuals in FPC’s nearby locations were transported fresh, a portion of some samples originating in FPC’s remote areas were transferred in potassium dichromate 2.5% to the processing location.

The sample size was calculated for unmatched Case–Control studies using Openepi software (https://www.openepi.com/Menu/OE_Menu.htm). To ensure a two-sided test with α = 0.05 and 80% power, a sample size of 44 (22 patients on corticosteroid therapy and 22 controls) with a percentage of controls with exposure (i.e. controls with assemblage A) of 45.7% [32] and odds ratio of 7 was required. The sample size was raised by 20% to account for probable dropout, missing data, and PCR-negative samples.

Thus, 52 microscopically positive faecal samples for *Giardia* cysts and/or trophozoites were collected as follows:

(i) Cases: *Giardia* positive – patients on corticosteroid therapy (POCT) (21 individuals) consisted of patients with any disease receiving active treatment with high dose corticosteroids (i.e., a course of ≥ 20 mg of prednisone per day when administered for at least two weeks). The preceding criteria were selected to accurately represent the status of immunocompromised according to the Center for Disease Control and Preventions (2023) [40] (Supplementary file, Table S1).
(ii) Controls: *Giardia* positive – patients (CONT) (31 individuals) comprised of individuals of any age, gender, not undergoing corticosteroid therapy, and who were in the absence of any underlying disease or impairment that affects physical, mental, and social well-being [41, 42]. The control individuals were included from those who were accompanying the patients (relatives and/or friends) and did not visit the hospital in search of medical advice. No current or previous parasitic infections were disclosed by the selected participants.

Patients who provided stool samples contaminated with urine or water, patients whose samples were too small (15–20 g—less than a full tablespoon), patients who were unsure of the dose and duration of corticosteroid treatment, or patients in the period of withdrawal from corticosteroid treatment were excluded from the study.

### Microscopic examination of the samples

Immediately upon receipt, the samples were separated into three portions:

i. A portion for wet mount and trichrome-stained smears were microscopically examined for *Giardia* cysts and trophozoites.
ii. A portion for the formalin ethyl acetate concentration technique, that was used to conduct additional wet mount and iodine microscopic examination.
iii. A portion for freezing at −20 °C, a dime-sized quantity of freshly acquired samples, and one milliliter of potassium dichromate-preserved samples.

**Fig. 1:**
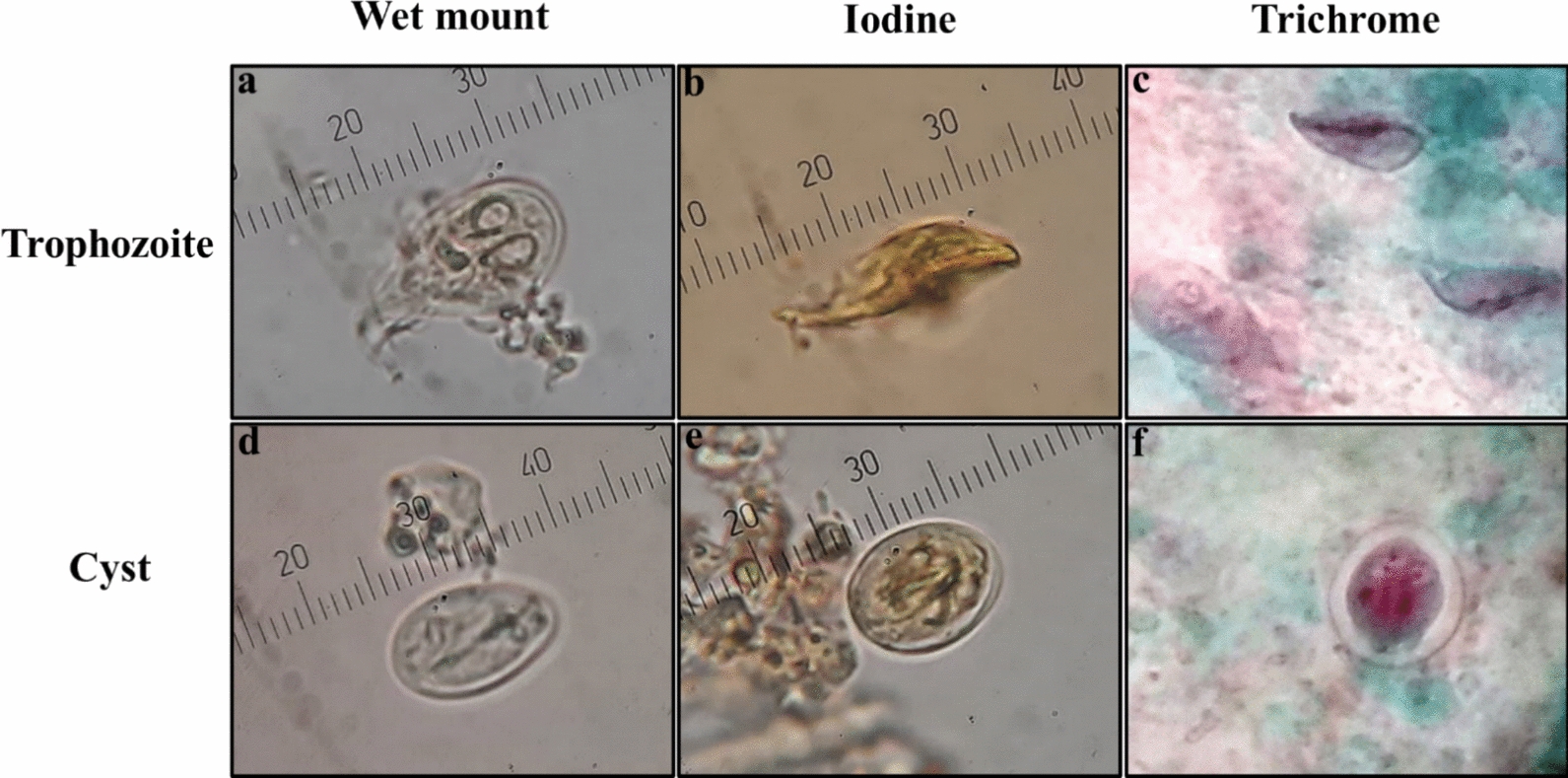
*Giardia duodenalis* trophozoites and cysts observed with a microscopic oil lens (× 1000). *Giardia* trophozoites’ appearance when examined in wet mount (**a**), stained with iodine (**b**), and trichrome stain (**c**). *Giardia* cysts examined in wet mount (**d**), stained with iodine (**e**), and trichrome stain (**f**)

Microscopically positive samples for *Giardia* (Fig. 1) were selected and sorted for further DNA extraction from the frozen samples. The microscopically positive *Giardia* samples were estimated positive by detecting *Giardia* cysts and/or *Giardia* trophozoites with a bright field microscopy and confirming the results with a trichrome-stained field.

Cysts were typically 11 to 14 µm in size and appeared ovoid to ellipsoid. Two and four nuclei were observed in different cysts exhibiting intracytoplasmic fibrils. The trophozoites, on the other hand were pear-shaped and ranged from 15 to 20 µm. Two anteriorly located nuclei and eight flagella were present in trophozoites, although they were rarely observed due to their weak staining. *Giardia* cysts and trophozoites can be plainly identified through wet mount, iodine, and trichrome staining (Fig. 1).

### DNA extraction

After being thawed with cold phosphate-buffered saline (PBS), the frozen faecal samples were filtered through layers of double gauze. To guarantee high-quality DNA devoid of impurities and inhibitors, repeated centrifugation (PLC-012E, 4,180 × g for 5 min) and washing with PBS (pH 7.4) were carried out until the supernatant turned clear [43, 44]. The supernatant was subsequently discarded, and by the manufacturer’s instructions, 200 µL of the 1 mL sediment-PBS was subjected to the InhibitEX lysis reagent from the Qiagen DNA Stool Mini Kit (Qiagen, Germany, GmbH). The procedure was marginally altered by adding 100 µL of elution buffer; the resultant DNA was subsequently chilled to −20 °C in preparation for further molecular analyses.

A Nanodrop lite Plus spectrophotometer (Thermo Scientific) was used to measure the sample absorbance at 260 nm in order to estimate the nucleic acid concentration. The absorbance ratios of 260/280 and 260/230 were utilized to calculate the purity of the DNA.

### Multi-locus sequence typing of ARE’s *Giardia duodenalis* isolates

The 52 Egyptian isolates underwent PCR amplification and sequencing analysis for *G. duodenalis* multi-locus genotyping.

#### Nested PCR amplification of *tpi*, *bg*, and *gdh* genes

Using previously published PCR procedures, partial coding sequences of three *Giardia* genes—*tpi*, *bg*, and *gdh*—were amplified in order to genotype *Giardia* isolates [34–38] respectively. Typically, 2 to 5 μL of sample DNA were used for the primary PCR, and 2 μL of the primary PCR product was applied to the nested or semi-nested PCR, depending on the original DNA concentration. Every PCR reaction was carried out in 25 μL volumes containing ten pmol of each primer and 12.5 μL of 2 × PCR TaqNova-Red PCR Mastermix (Blirt, RP85T) with a final MgCl_2_ concentration of 2 mM. In addition, a minor modification was implemented by incorporating 1 μL of bovine serum albumin (BSA 10 mg/ml) in each primary PCR reaction, improving the amplification yield. Each PCR reaction was performed in duplicate, and DNA purified from the fecal material of an animal positive for *G. duodenalis* was used as a control.

The *G. duodenalis tpi*, *bg*, and *gdh* genes were amplified using the PCR methods indicated in Table 1 utilizing previous PCR procedures [45–50] with some adjustments to boost the PCR yield of some weak bands. In samples that were negative for *Giardia*-PCR, templates with minimal copy counts were observed. Therefore, the inhibition test and sample dilution were not performed. However, the PCR conditions were altered. When the PCR was repeated, some of the negative samples yielded results, while others remained negative. The PCR results were confirmed using agarose gel (1%) electrophoresis, and they were purified by following the manufacturer's instructions with the ExtractMe^®^ DNA Clean-up Gel-out kit (Blirt, Gdańsk, Poland). Purified DNA from a goat excrement sample that was positive for *Giardia* was used as the positive control, while sterile PCR-grade water was used as the negative control.

**Table 1:** PCR analysis for the amplification of *tpi*, *bg*, and *gdh* genes of *Giardia duodenalis*

Gene/Locus	PCR type	Primers code	Primers structure	PCR system*		Final PCR Product	Ref.
*Tpi*	1^ry^	AL3543	5'-AAATIATGCCTGCTCGTCG-3'	Initial denaturation	95 °C / 5 min	530 bp	[34]
		AL3544	5'-CAAACCTTITCCGCAAACC-3'	40 cycles	95 °C / 45 s		
	2^ry^	AL3544	5'-CCCTTCATCGGIGGTAACTT-3'		50 °C / 1 min		
		AL3545	5'-GTGGCCACCACICCCGTGCC-3'		72 °C / 1 min		
				Final extension	72 °C / 7 min		
*Bg*	1^ry^	G7F	5’-AAGCCCGACGACCTCACCCGCAGTGC-3’	Initial denaturation	95 °C / 5 min	511 bp	[35, 36]
		G759R	5’-GAGGCCGCCCTGGATCTTCGAGACGAC-3’	40 cycles	95 °C / 30 s		
					63 °C / 1 min		
					72 °C / 1 min		
				Final extension	72 °C / 7 min		
	2^ry^	GBF	5’-GAACGAACGAGATCGAGGTCCG-3’	Initial denaturation	95 °C / 15 min		
		GBR	5’-CTCGACGAGCTTCGTGTT-3’	40 cycles	95 °C / 30 s		
					55 °C / 1 min		
					72 °C / 1 min		
				Final extension	72 °C / 7 min		
*Gdh*	1^ry^	GDHeF	5’-TCAACGTYAAYCGYGGYTTCCGT-3’	Initial denaturation	95 °C / 5 min	432 bp	[37, 38]
		GDHiR	5’-GTTRTCCTTGCACATCTCC-3’	40 cycles	95 °C / 30 s		
	Semi-nested	GDHiF	5’-CAGTACAACTCYGCTCTCGG-3’		56 °C / 1 min		
		GDHiR	5’-GTTRTCCTTGCACATCTCC-3’		72 °C / 1 min		
				Final extension	72 °C / 7 min		

^*^The PCR systems have been adjusted to boost the PCR yield of some weak bands. *Tpi*: Triosephosphate isomerase; *bg*: β-giardin; *gdh*: Glutamate dehydrogenase; 1^ry^: Primary; 2^ry^: Secondary; Ref.: Reference; min: Minutes; s: Seconds; bp: Base pairs

#### *G. duodenalis* sequencing and assemblage identification

Using the forward starter, the amplicons were sequenced in a unidirectional manner. Raw sequencing output was analyzed in Geneious Prime 2023.2.1 (https://www.geneious.com/). After trimming the ends, base calls were inspected manually, with ambiguities introduced following the recommendations of the International Union of Pure and Applied Chemistry [51].

The species were confirmed, and assemblages were identified by querying GenBank with post-processed reads using Basic Local Alignment Search Tool (BLAST). For each sequence, the top match with assemblage annotation was selected. The obtained sequences were supplemented with records of known sub-assemblage retrieved from GenBank, aligned by MUSCLE algorithm and trimmed to equal lengths in MEGA11 (https://www.megasoftware.net/) [52].

In order to construct a Maximum Likelihood (ML) tree, the best replacement model for each alignment was determined and chosen using the Bayesian Information Criterion and the Akaike Information Criterion. Bootstrapping was used to calculate branch support (1000 replicates).

### Statistical analysis

Descriptive statistics are shown as frequencies (categorical variables) or mean with standard deviation (SD) (continuous variables).

Individual variables were evaluated as possible risk factors by determining their odds ratio (OR) and 95% confidence intervals (CIs). Cross-tabulations were created, and either Pearson’s chi^2^ test or Fisher’s exact test was applied. Similarly, a T-test was used for mean age comparison. The statistical analyses were carried out using Stata software, version 16 (StataCorp, College Station, TX, US).

## Results

### Characteristics of POCT and CONT individuals

Overall, 52 individuals infected with *G. duodenalis* were included: 21 POCT and 31 CONT individuals. The majority were males (59.6%), the mean age was 12.3 years and resided in rural areas (73.1%). The POCT group had a lower proportion of male participants, and a higher mean age compared to the CONT group (38.1% vs 74.2%; OR = 0.2; p = 0.009 and 21.7 vs 5.8 years; OR = 1.5; p = 0.006, respectively) (Table 2).

**Table 2:** Participants characteristics and risk factor analysis between patients on corticosteroid therapy (POCT) and controls (CONT)

Variable	Categories	Total (N = 52)		POCT (N = 21)		CONT (N = 31)		OR	95% CI	p-value
		N	%	N	%	N	%			
Sex	Male	31	59.6	8	38.1	23	74.2	0.2	0.1–0.8	**0.009**
	Female	21	40.4	13	61.9	8	25.8	Ref		
Mean age in years (SD)		12.3 (16.7)		21.7 (23.4)		5.8 (1.9)		1.5	1.1–2.1	**0.006**
Residence	Urban	14	26.9	0	0.0	14	45.2	NC		
	Rural	38	73.1	21	100.0	17	54.8	Ref		
Symptomatic	Yes	25	48.1	9	42.9	16	51.6	0.7	0.2–2.5	0.535
	No	27	51.9	12	57.14	15	48.4	Ref		
Abdominal pain	Yes	18	72.0	6	28.6	12	38.7	0.7	0.1–6.2	0.673
	No	7	28.0	3	14.3	4	12.9	Ref		
Diarrhoea	Yes	10	40.0	6	28.6	4	12.9	6.0	0.8–52.6	0.087
	No	15	60.0	3	14.3	12	38.7	Ref		
Other symptoms*	Yes	10	40.0	9	42.9	1	3.2	NC		
	No	15	60.0	0	0.0	15	48.4	Ref		
Owns domestic animals	Yes	21	40.4	6	28.6	15	48.4	0.4	0.1–1.6	0.153
	No	31	59.6	15	71.4	16	51.6	Ref		
Has access to potable water	Yes	45	86.5	21	100.0	24	77.4	NC		
	No	7	13.5	0	0.0	7	22.6	Ref		
Has access to sewage system	Yes	39	75.0	15	71.4	24	77.4	0.7	0.2–3.2	0.625
	No	13	25.0	6	28.6	7	22.6	Ref		
Concomitant parasitic infection	Yes	17	32.7	15	71.4	2	6.5	36.3	5.6–367.7	** < 0.001**
	No	35	67.3	6	28.6	29	93.5	Ref		

^*^Other symptoms refer to epigastric pain, postprandial heartburn, constipation, loss of weight, nausea, and pallor

Bold numbers reflect statistically significant association

*OR* Odds ratio, *CI* Confidence interval, *Ref* Reference category, *SD* Standard deviation, *POCT* Patients on corticosteroid therapy, *CONT* Controls, *NC* Not calculable (Zero observations in cells)

Among the participants, symptoms were reported by almost half (48.1%), with the majority experiencing abdominal pain (72%), followed by diarrhea (40%) and other symptoms (epigastric pain, postprandial heartburn, constipation, loss of weight, nausea, pallor). The proportion of symptoms did not differ between POCT and CONT individuals. Among those with symptoms, the majority (76%, N = 19) had a single infection of *Giardia*, while a smaller proportion (24%, N = 6) had mixed parasitic infection (Table 3). However, the association between symptomatology status and presence of concomitant infections was not statistically significant.

**Table 3:** Concomitant parasitic infections associated with *Giardia duodenalis* in patients on corticosteroid therapy (POCT) and controls (CONT)

Variable	Categories	POCT (21)	CONT (31)	Asymptomatic (27)	Symptomatic (25)
No. of parasitic infections	Single *Giardia* infection	6	29	16	19
	Double	8	2	7	3
	Triple	4	0	3	1
	Quadruple	2	0	1	1
	Quintuple	1	0	0	1
Concomitant parasitic infection		15	2	11	6
	*Blastocystis* sp.	1	2	2	1
	*Entamoeba coli*	7	0	5	2
	*Chilomastix mesnili*	4	0	1	3
	*Hymenolepis nana*	2	0	1	1
	*Enterobius vermicularis*	1	0	0	1
	*Dientamoeba fragilis*	7	0	6	1
	*Endolimax nana*	4	0	1	3

*POCT* Patients on corticosteroid therapy, *CONT* Controls, *No.* Number, *sp.* Species

Most participants (59.6%) own domesticated animals, while 86.5% have access to a water supply and 75.0% have a sewage system in their households; there were no differences between the two groups for these exposures.

Notably, 67.3% of patients had a single infection, with the POCT group having 36 times higher probability of having concomitant parasitic infection, compared to the CONT group (OR = 36.3; p < 0.001) (Table 2). Concomitant parasitic infection was the predominant type in the POCT group, with up to quintuple parasites identified, while most CONT group had a single type of infection. Concomitant parasitic infections were detailed and reported (Table 3).

### Assemblages’ identification of *Giardia duodenalis* in the POCT and CONT

*Giardia* DNA was detected with at least one marker in 38 samples (73%), 15 POCT (71%) and 23 CONT (74%) (Supplementary File, Figure S1). Twelve samples were typed at all three loci, eleven at two loci, and fifteen at one locus (Table 4). PCR was repeated for negative samples, but the prevalence did not increase; if a sample was negative on the first attempt, PCR for each marker was repeated at least once.

**Table 4:** *Giardia duodenalis* assemblages detected in patients on corticosteroid therapy (POCT) and controls (CONT)

No.	Group	Serial	Symp. Assoc.	*tpi*	*bg*	*gdh*	Assemblage detected	Positive/total
1	POCT	A21	1	―	―	B	B	15/21
2	POCT	A22	1	―	―	―	―	
3	POCT	A23	2	―	―	―	―	
4	POCT	A24	2	―	―	B	B	
5	POCT	A25	2	B	―	―	B	
6	POCT	A26	2	―	―	―	―	
7	POCT	A27	2	―	―	B	B	
8	POCT	A28	1	A	―	―	A	
9	POCT	A29	2	A	―	―	A	
10	POCT	A30	1	―	―	AII	A	
11	POCT	A31	1	―	―	―	―	
12	POCT	A32	2	A	―	B	A + B	
13	POCT	A33	2	A	―	―	A	
14	POCT	A34	2	A	―	B	A + B	
15	POCT	A35	2	A^a^	―	―	A	
16	POCT	A36	2	―	―	AII	A	
17	POCT	A37	1	―	―	―	―	
18	POCT	A38	1	B	―	―	B	
19	POCT	A39	1	A	―	B	A + B	
20	POCT	A40	1	―	―	―	―	
21	POCT	A41	2	A	―	―	A	
22	CONT	B01	1	―	B	AII	A + B	23/31
23	CONT	B02	1	―	―	―	―	
24	CONT	B03	1	B	B	B	B	
25	CONT	B04	1	―	―	―	―	
26	CONT	B05	1	―	―	―	―	
27	CONT	B06	1	A	―	―	A	
28	CONT	B07	1	―	―	―	―	
29	CONT	B08	1	―	―	―	―	
30	CONT	B09	1	―	―	―	―	
31	CONT	B10	1	―	―	―	―	
32	CONT	B11	1	―	―	―	―	
33	CONT	C12	2	A	AII	AII	A	
34	CONT	C13	2	B	B	B	B	
35	CONT	C14	2	B	B	AII	A + B	
36	CONT	C15	2	A	B	―	A + B	
37	CONT	C16	2	B	AII	―	A + B	
38	CONT	C17	2	B	B	E	B + E	
39	CONT	C18	2	B	B	―	B	
40	CONT	C19	2	B	B	―	B	
41	CONT	C20	2	B	B	B	B	
42	CONT	D42	1	―	B	E	B + E	
43	CONT	D43	1	―	B	―	B	
44	CONT	D44	1	A	AII	―	A	
45	CONT	D45	2	―	B	―	B	
46	CONT	D46	2	B	B^a^	B	B	
47	CONT	D47	2	B	B^a^	AII	A + B	
48	CONT	D48	2	B	B	B	B	
49	CONT	D49	2	B	B	B	B	
50	CONT	D50	1	B	B	B	B	
51	CONT	D51	1	B	B^a^	E	B + E	
52	CONT	D52	2	A	B	―	A + B	
Total				29/52	22/52	22/52	38/52	

^a^Sequence excluded from the alignment due to poor coverage; ―: Not amplified

*POCT* Patients on corticosteroid therapy, *CONT* Controls, *Symp. Assoc*. Symptoms association, *1* Symptomatic, *2* Asymptomatic

The PCR targets *tpi*, *gdh* and *bg* were meticulously amplified and successfully sequenced for 29, 22 and 22 isolates, respectively, ensuring the reliability of data. It is worth noting that fourteen samples failed to produce positive results in PCR, although they were microscopically positive (Fig. 1; Supplementary File, Figure S1).

In summary, the obtained sequences were carefully analyzed and submitted to GenBank (***tpi*:** PP566746-PP566774; ***bg*:** PP566775-PP566796; ***gdh*:** PP576000-PP576021) (Supplementary File, Tables S3–S5), contributing to the growing knowledge on *Giardia* genetic variation.

BLAST analysis revealed representatives of assemblage A, B and E (Supplementary File, Tables S3–S5). Due to the differences in sequence coverage, samples A35 (*tpi*), D46 (*bg*), D47 (*bg*), and D51 (*bg*) were excluded from alignments to maximize the amount of genetic variation analyzed (Table 4).

Sixteen isolates (42.0%) were identified as assemblage B and ten isolates (26.3%) as assemblage A. In contrast, twelve *G. duodenalis* isolates (31.6%) exhibited discordant assignments indicating mixed infections. Based on the *gdh* gene, assemblage E was detected in three isolates. Assemblage-B *bg* and assemblage-E *gdh* co-occurred (Table 4).

Assemblage B predominated among controls, whereas assemblage A was the most frequent variant among patients on corticosteroid therapy.

### Factors associated with *Giardia duodenalis* genotypes

**Fig. 2:**
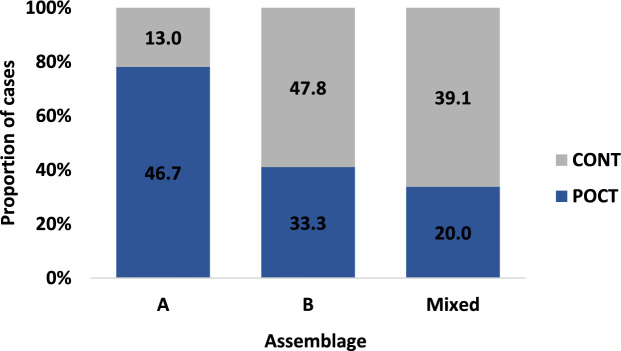
Distribution of *Giardia* assemblages by cases and controls. *POCT* Patients on corticosteroid therapy, *CONT* Controls

The POCT group appeared to have a higher likelihood of being infected with *G. duodenalis* assemblage A, rather than mixed assemblage, compared to the CONT group, despite the association not reaching statistical significance (OR = 7.0; p = 0.084). (Table 5, Fig. 2).

**Table 5:** *Giardia* infection assemblages in patients on corticosteroid therapy (POCT) and control (CONT) groups (univariate analysis).

Assemblage	Total (N = 38)	POCT (N = 15)	CONT (N = 23)	OR	95% CI	p-value
	N (%)	N (%)	N (%)			
A	10 (26.3)	7 (46.7)	3 (13.0)	7.0	0.8–68.6	0.084
B	16 (42.1)	5 (33.3)	11 (47.8)	1.4	0.2–11.1	1.000
Mixed^a^	12 (31.6)	3 (20.0)	9 (39.1)	Ref		

^a^Mixed A + B or B + E

*POCT* Patients on corticosteroid therapy, *CONT* Controls, *OR* Odds ratio, *CI* Confidence interval, *N* Number

The present research revealed an interesting pattern among symptomatic individuals, with an equal distribution of infections among assemblage A (*n* = 4), B (*n* = 5), and mixed (*n* = 5). In contrast, asymptomatic patients showed a higher prevalence of assemblage B (*n* = 11), followed by mixed (*n* = 7) and assemblage A (*n* = 6). However, no statistically significant differences were detected between *G. duodenalis* assemblages and symptomatology. Similarly, no significant differences were identified between *G. duodenalis* assemblages and other factors (i.e. residence, animal ownership, access to water, prior infection) (Supplementary File, Table S2).

### Phylogenetic analysis of *Giardia duodenalis* assemblages / sub-assemblages in POCT and CONT

**Fig. 3:**
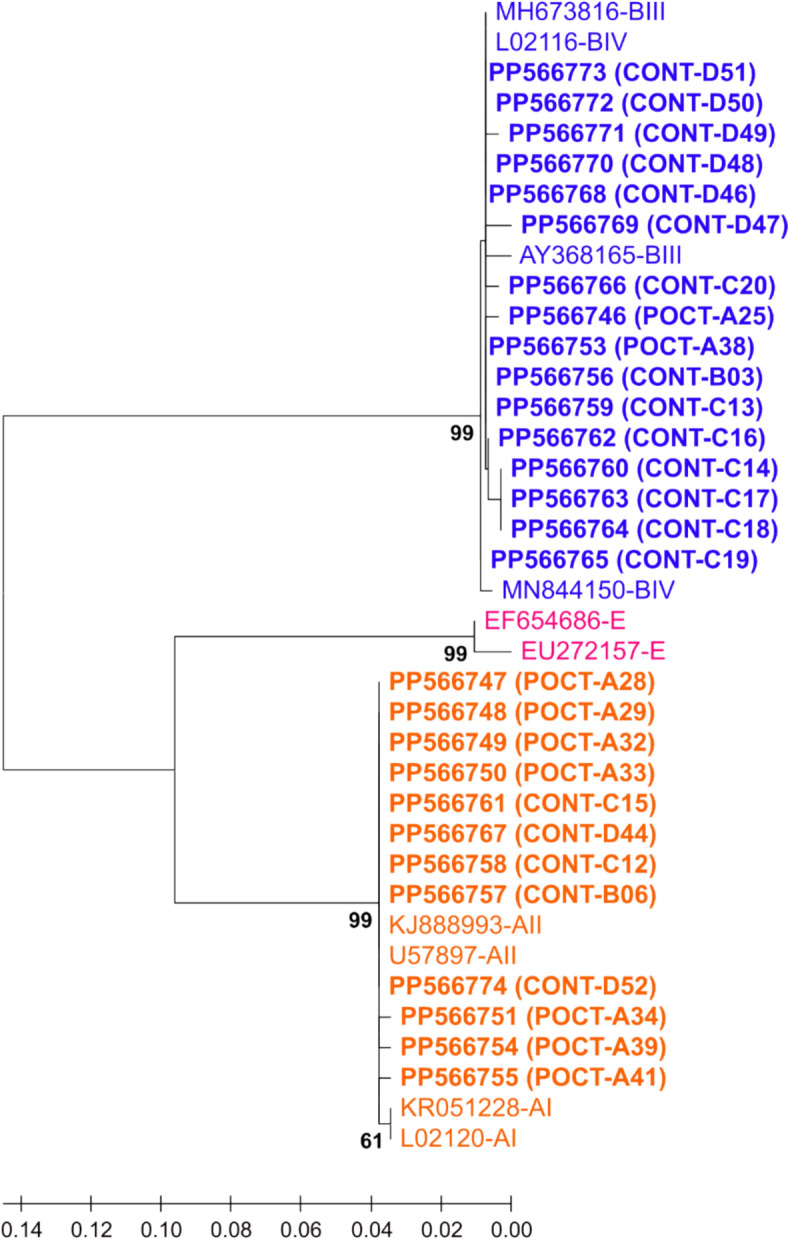
Maximum Likelihood (ML) unrooted tree with the highest log likelihood (−804.94), based on partial sequences of the *tpi* gene of *Giardia duodenalis*, generated using the Kimura 2-parameter model (K2P) [53]. *G. duodenalis* assemblages of the current study were colored with three distinct colors: Orange for genotype A, blue for genotype B and pink for genotype E. The dataset comprised 38 sequences: 28 sequences of *tpi* gene obtained in this study (A25-D52, Bold) were compared with ten reference sequences of known sub-assemblage from GenBank. Next to the branches, the proportion of trees (1000 replicates) in which the related taxa clustered together is displayed. Entire bootstrap value > 50% is displayed. The branch lengths of the scaled-up tree are expressed in terms of the number of substitutions made at each site. There were 283 positions in the final dataset. POCT: Patients on corticosteroid therapy; CONT: Controls

**Fig. 4:**
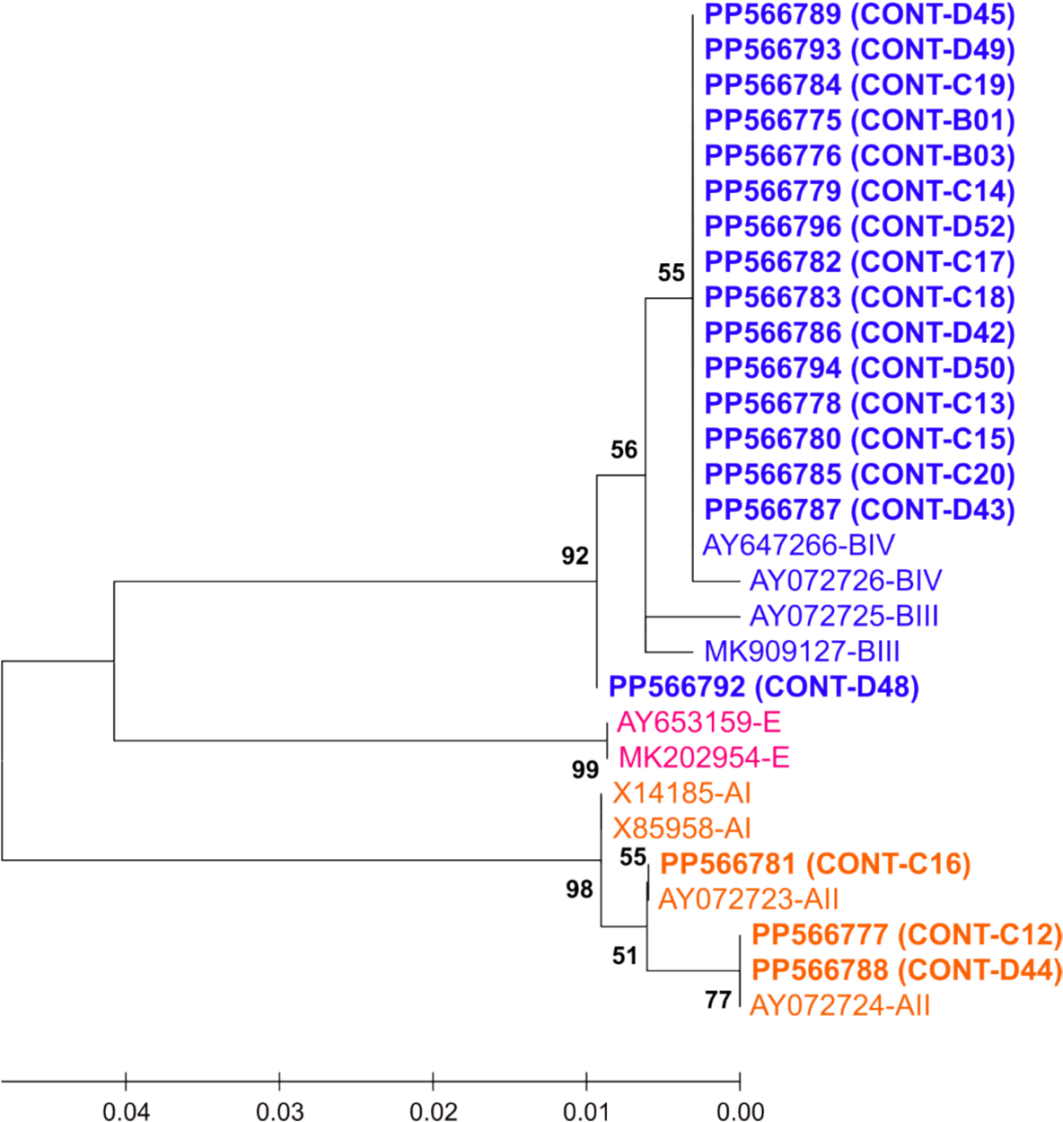
Maximum Likelihood (ML) unrooted tree with the highest log likelihood (−648.64), based on partial sequences of the *bg* gene of *Giardia duodenalis*, generated using the Tamura-Nei substitution model (TrN) [54]. *G. duodenalis* assemblages of the current study were coloured with three distinct colours: Orange for genotype A, blue for genotype B and pink for genotype E. The dataset comprised 29 sequences: 19 sequences of *bg* gene obtained in this study (B01-D52) were compared with ten reference sequences of known sub-assemblage from GenBank. Next to the branches, the proportion of trees (1000 replicates) in which the related taxa clustered together is displayed. Entire bootstrap value > 50% is displayed. The branch lengths of the scaled-up tree are expressed in terms of the number of substitutions made at each site. There were 327 positions in the final dataset. POCT: Patients on corticosteroid therapy; CONT: Controls.

**Fig. 5:**
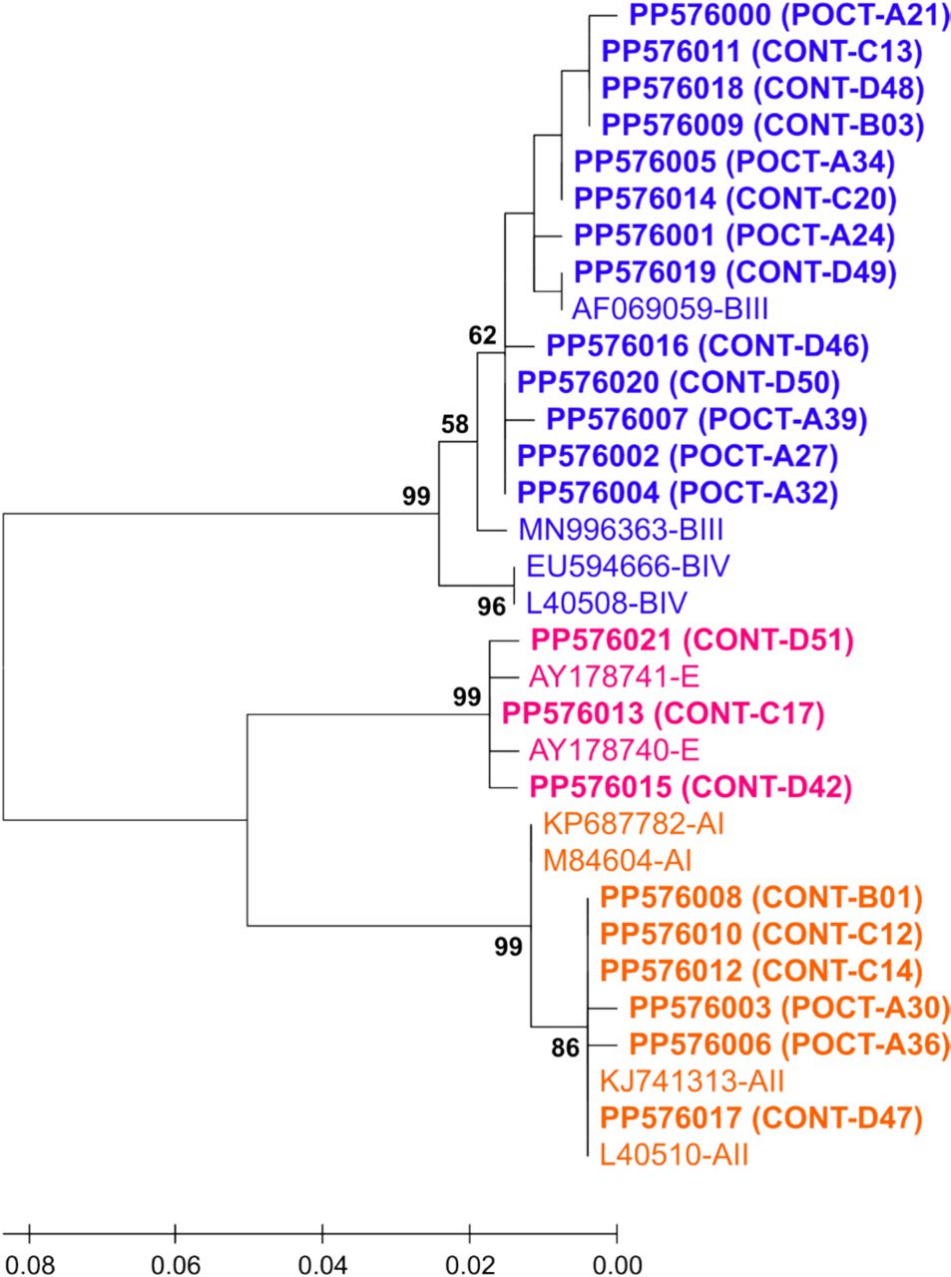
The maximum likelihood (ML) unrooted tree with the highest log likelihood (−675.06) was produced using the Tamura 3-parameter substitution model (T92) [54] and was based on partial sequences of the *Giardia duodenalis gdh* gene. *G. duodenalis* assemblages of the current study were colored with three distinct colors: orange for genotype A, blue for genotype B and pink for genotype E. The dataset comprised 32 sequences: 22 sequences of *gdh* gene obtained in this study (A21-D51, Bold) were compared with ten reference sequences of known sub-assemblage from GenBank. Next to the branches, the proportion of trees (1000 replicates) in which the related taxa clustered together is displayed. Entire bootstrap value > 50% is displayed. The rate variation model allowed for some sites to be evolutionarily invariable (40.82% sites). The tree is drawn to scale, with branch lengths measured in the number of substitutions per site. There were 267 positions in the final dataset. *POCT* Patients on corticosteroid therapy, *CONT* Controls

Maximum Likelihood (ML) unrooted trees with the highest log likelihood, based on partial sequences of the three gene loci of *G. duodenalis* (Figs. 3, 4, 5, Supplementary File Tables S3-S5), were successfully generated using the Kimura 2-parameter model (K2P) [53]. The three phylogenetic trees contained sequences of the current study compared with ten DNA reference sequences chosen based on assemblages / sub-assemblages GenBank record or relevant articles.

In all three Maximum Likelihood trees (Figs. 3, 4, 5), *G. duodenalis* assemblage clusters A, B and E emerged with strong bootstrap support (B = 92–99%). The *G. duodenalis* assemblages of the current study and reference sequences were coloured with orange for assemblage A, blue for assemblage B and pink for assemblage E.

Partial coding sequences of the *tpi* (283 bp) and *bg* genes (327 bp) formed assemblages A and B (Figs. 3 and 4). A moderate support of 77% in the *bg* tree implicated that samples C12 and D44 belong to sub-assemblage AII (Fig. 4). The *gdh* tree confirmed the assignment of samples C17, D42 and D51 to assemblage E; all assemblage-A samples clustered with sub-assemblage AII reference sequences (B = 86%) (Fig. 5).

## Discussion

The Arab Republic of Egypt is classified as a low-to middle-income nation, where *Giardia* is a prevalent pathogen, with prevalences ranging from 21 to 50% [55, 56]. This study aimed to analyze the molecular and epidemiological data of *G. duodenalis* in both immunocompromised and immunocompetent groups, focusing on risk factors for giardiasis in Ismailia. It was hypothesized that the association of risk factors with specific *Giardia* assemblage might vary between these groups.

Patients undergoing corticosteroid therapy showed a significantly higher likelihood of concomitant infections compared to those who were not treated with corticosteroids (OR = 36.3; p < 0.001). This strong correlation emphasizes the clinical importance of screening immunocompromised individuals for mixed parasitic infections. In the POCT group, concomitant parasitic infection predominated, with up to five parasites identified, whereas the CONT group mostly had  a single infection. Individuals with compromised immune systems like POCT are at an increased risk of contracting parasitic, bacterial, viral, and fungal infections, which are generally eliminated by those with a healthy immune system. The POCT group, as a category of immunocompromised [41] characterized by impaired cellular and humoral responses and reduced T and B lymphocyte activity, is more prone to complications from minor infections and concurrent infections [57, 58]. Similarly, individuals receiving cortisone medication in Ismailia were found to have mixed opportunistic parasite infections, with a notable correlation between *G. duodenalis* and *Cryptosporidium* sp. infections [39]. Mixed opportunistic parasite infections were also prevalent among immunocompromised patients in Cairo, particularly those with diabetes, cancer and renal transplants [33]. Likewise, concomitant infections have been reported in immunocompromised patients in Egypt and Yemen [58, 59]. In Sohag, hemodialysis patients with a compromised immune system had a significantly higher incidence of mixed parasitic infections compared to the control group [60]. Patients on corticosteroid therapy have been reported to develop hyper-infection and dissemination of *Strongyloides stercoralis*, and in severe cases, resulted in mortality [61]. Unlike protozoan infections, helminth infections are more exacerbated due to corticosteroids effects on immune pathways crucial for helminth immunity.

Over half of *Giardia*-positive individuals (GPI) (52%) in this study were asymptomatic. Among symptomatic patients, abdominal pain and diarrhea were the most common symptoms. *G. duodenalis* infection did not correlate with symptoms in the POCT and CONT groups. Immunocompetent individuals typically experienced self-limiting infections, whereas immunocompromised individuals are at higher risk for severe *Giardia* infections. Refractory giardiasis has been reported in patients with immunosuppression, such as hypogammaglobulinemia and nephrotic syndrome [12, 13]. Cancer patients were reported to be 1.24 times more likely to contract a *G. duodenalis* infection than healthy controls [14]. Chronic diarrhoea over a 6-month period due to giardiasis was documented in renal transplant patients [15]. *In vivo*, dexamethasone increased *Giardia* parasitic load and intestinal permeability in gerbils [26]. Patients on steroids may exhibit less pronounced infection symptoms due to reduced cytokine release and inflammatory response, delaying infection early detection [62]. Asymptomatic carriers contribute significantly to *Giardia* transmission, even without symptoms. Diarrhoea and recurrent abdominal pain are primary symptoms associated with giardiasis, although acute presentations are often attributed to other causes. Symptom severity is closely linked to parasite virulence, host nutritional status, developmental stage, and immunological conditions [63–66].

It is interesting to note that approximately 40% of the GPI are engaging in animal husbandry and 73% reside in rural areas. Animal husbandry is also a common practice in urban areas. The governorate of Ismailia, apart from its urban core, consists of six rural municipalities. In most rural and agricultural regions of Ismailia, domesticated animals are a household staple. When hygiene is insufficient—characterized by poor sanitation, overcrowding, and low socioeconomic conditions—there is an increased risk of parasites being transmitted between humans and animals, and vice versa [67]. There have been reports of zoonotic transmission, where humans and animals share the same *Giardia* genotypes [27, 28, 68].

Some participants in the present study lacked access to clean drinking water (7 individuals, 13.4%) and sewage disposal (13 individuals, 25%). Despite water, sanitation and hygiene (WASH) initiatives, in collaboration with the Water Supply and Sanitation Collaborative Council (WSSCC), over 50% of rural Egypt lacks sewage systems [69, 70] facilitating waterborne disease transmission and air pollution from raw sewage. Limited access to fresh water and the need to purchase and transport water are common in rural West Ismailia, linked to *Blastocystis* sp. infection [67]. Indiscriminate defecation near water sources of Ismailia is common in areas with inadequate sewage facilities. Lack of sewage disposal has been reported to increase the risk of *Giardia* and mixed parasitic infections [71–73].

Of the microscopically positive samples, 38 of 52 GPI were identified molecularly. Positive *Giardia* results were obtained when at least one target gene was amplified. Negative PCR samples, despite repeated testing, showed no or faint results. The exclusion of these isolates is a study limitation; however, PCR-negative samples were evenly distributed across POCT and CONT groups, reducing potential bias. Larger sample sizes, therefore, could improve robustness. Previous studies in Egypt, Kenya, and Brazil reported negative PCR results for positive *Giardia* microscopic samples [32, 74–76]. However, the negative PCR result in this study could be ascribed to: (i) The PCR yield, which may be influenced by the DNA inhibitors present in the stool samples and the DNA extraction method or reagents utilized; (ii) Method of sample preservation: the authors observed that samples preserved in K dichromate yielded a weaker PCR signal than samples preserved freshly; (iii) Variations in PCR amplification parameters: it is observed that distinct amplification conditions generated distinct yields; for instance, increasing the number of PCR cycles to 40 and incorporating bovine serum albumin (10 mg/mL) in the primary PCR increased the yield. Molecular methods are gaining prominence in research and diagnostics; they are widely regarded as the most precise and sensitive tests for routine surveillance and diagnosis of *Giardia* infection [77–79]. Nevertheless, in communitites with limited resources, microscopy will continue to be a dependable diagnostic method if performed by an experienced microscopist [75, 80, 81].

Using multiple gene loci in PCR-based diagnosis of giardiasis is advantageous due to varying discriminatory abilities of these genes. Employing two gene loci with greater polymorphism (*tpi* and *gdh*), along with more conserved locus (*bg*), enhances the test's sensitivity [82]. In this study, some samples experienced false negative amplification at certain loci. It was also noted that there were differences in sensitivity and bias among the three loci when amplifying a specific assemblage, resulting in inconsistent outcomes for some participants. Similar observations have been made by other researchers [27, 29, 34, 83]. Due to the significant genetic diversity among *G. duodenalis* isolates [83], inadequate primer specificity might lead to amplification failure at a particular locus. Single-nucleotide polymorphisms, insertion-deletions, and various *Giardia* genotypes are contributing factors [32].

Our research indicates that the *tpi* gene was the most successful among the three markers studied. It has been noted that the *tpi* gene can distinguish specific isolates that other loci cannot [47], which was also observed in this study. The *tpi* gene is a reliable phylogenetic marker for analyzing the taxonomic and molecular evolutionary relationships within the species *G. duodenalis* [47].

The molecular data from this analysis revealed that the prevalence of assemblages A and B differed significantly in the Ismailia population, with assemblage B being the most common, followed by assemblage A/AII. Mixed infections of assemblages A + B accounted for one-third of the amplified isolates, and three isolates showed mixed infections with assemblages B + E. Several studies conducted in Egypt across different governorates also concluded that assemblage B was more prevalent than other assemblages (Supplementary File, Table S6). However, a limited number of surveys and reports from other African regions provided evidence suggesting that assemblage A was more widespread than assemblage B [31, 32, 84, 85]. Mixed infections involving *Giardia* assemblages/sub-assemblages (A + B, A + E, and B + E) have been documented in various Egyptian studies involving both immunocompetent and immunocompromised individuals using multiple genetic loci (Supplementary File, Table S6). In Brazil, four *Giardia* assemblages—A, B, C, and D—were identified in immunocompromised patients after chemotherapy [86]. In China, HIV patients were found to have *Giardia* assemblages C, B, and mixed B + C [87]. In Iran, *Giardia* infections with AI, AII genotypes, and mixed (AI + B) infections were observed in cancer and HIV patients [88]. The presence of mixed infections in this and previous studies may indicate the existence of two genetically distinct assemblages, with one assemblage preferentially amplified at a specific locus over the other. Mixed assemblages’ infections in both immunocompetent and immunocompromised individuals in this study suggest different transmission routes. The variations in the geographic distribution of *G. duodenalis* genotypes may reflect different infection sources and transmission pathways [83].

Genetic exchanges between assemblages within a single *Giardia* cyst can lead to mixed infections [89]. Hashemi-Hafshejani et al. (2022) [25] developed a specific set of *tpi*-mixed primers to detect these mixed infections. Their research found that samples showing assemblage A or B across three loci also displayed A + B in the *tpi*-mixed test. Some samples might have been misidentified as solely A or B without this primer set. This suggests that some unanimous assignments in our study could be mixed. Sanger sequencing, which provides only one read per sample, cannot detect multiple variants within a single amplicon. Next-generation sequencing or cloning and sequencing of PCR products would be advantageous in such situations. Similar findings were reported by Messa et al. (2021) [83] and Helmy et al. (2014) [28], who noted that ambiguous sequences, particularly those linked to mixed infections, need further investigation. The variability in *Giardia* assemblage typing may be due to differences in the resolution power of gene loci and the substitution rates at various genetic loci [90]. The complexity of mixed assemblage infections might reflect increased infection pressures from *Giardia* parasites and offer insights into the parasite's epidemiological status [29].

Assemblage E was detected in three samples in this study (i.e., B + E). Two of these samples were from individuals who did not report owning domestic animals, and two were from individuals living in rural areas. However, the sample size for this subgroup needs to be more significant to conduct a meaningful correlation analysis, necessitating further investigation. Gene sequencing has identified assemblage E in the faeces of various domesticated animals, including cattle, sheep, rodents, rabbits, and yaks [91–94]. Assemblage E, typically linked to animals in human samples, poses significant public health concerns. Studies in Egypt have associated assemblage E with rural settings, low settings, low-income areas, and cattle farming [27, 28, 30, 40]. Abdel-Moein and Saeed (2016) [29] found that livestock-specific assemblage E was prevalent among humans and calves in the same area in Cairo. Foronda et al. (2008) [31] reported that assemblage E made up 15% of the positive human samples in Egypt. In the governorate of Ismailia, assemblage E was found in humans and livestock at the same location [27]. It was the most common assemblage, found in 13% of calves in Mansoura [95]. The ability of animal assemblages to cross species barriers and infect humans highlights the need for further research into the prevalence of *G. duodenalis* assemblages other than A and B in humans. Future research should concentrate on zoonotic transmission mechanisms and intervention strategies in rural Egyptian communities.

The current study suggests a potential link between *G. duodenalis* assemblage A in the POCT group, compared to the CONT group although this association was not statistically significant (p = 0.084). Assemblage A has been associated with iron deficiency anaemia and diarrhoea in Egyptian children [34, 96, 97]. It was also connected to recurrent *Giardia* infection and mixed assemblages in Brazil [94]. Binary logistic regression showed a link between assemblage A and stomach upset in Kenya [98]. Assemblage A was the most common genotype in colorectal cancer cases [99]. In contrast, assemblage B has been associated with HIV infection [100] and was positively correlated with asymptomatic HIV-positive Kenyan children [101] and Chinese HIV patients [87]. *In vitro* studies showed that assemblage A grows faster, encysts/excysts more efficiently, and causes more tissue damage and intestinal microbiota abnormalities in mice compared to assemblage B [102]. The association of assemblage A with POCT remains uncertain. Previous studies could not determine whether an immunocompetent could tolerate an infection with a specific *Giardia* genotype, whereas an immunocompromised could not. Further research with a larger sample size is needed to explore this question.

In this study, symptoms were not linked to any specific assemblage. Regardless of the assemblage, symptoms might be related to the virulence factors of the *Giardia* parasite [103]. Even though assemblages A and B are regarded as the most virulent [103], it is challenging to identify common virulence factors and establish a connection between symptoms and assemblages. This difficulty arises from the presence of asymptomatic carriers and significant genetic diversity within and between these assemblages.

## Conclusion

The current study enhances understanding of giardiasis epidemiology in the ARE and highlights the unique patterns of assemblages that differentiate patients on corticosteroid therapy from control groups. In patients receiving corticosteroid therapy, there appears to be a potential link with *G. duodenalis* assemblage A. These patients are also more prone to concurrent parasitic infections. The presence of sub-assemblage AII suggests anthroponotic transmission of giardiasis in this study group. Furthermore, detecting assemblage E and mixed infections indicates possible zoonotic transmission.

Comprehensive clinical-epidemiological studies with larger sample sizes are needed to determine if the clinical progression of *G. duodenalis* infection indeed varies among different assemblages, clusters, and transmission routes. This can be facilitated by using assemblage information for surveillance. Equally important, is the need to implement better preventive measures for giardiasis. Increasing public awareness about infection transmission, especially among rural populations and those undergoing corticosteroid therapy, is a key step in this direction. Additional research with more faecal samples and next-generation sequencing using extra markers is required to understand the extent of giardiasis in the local animal and human host diversity and transmission dynamics in the ARE population.

## Supplementary Information

Additional file 1

## Data Availability

No datasets were generated or analysed during the current study.
