# Correction: Molecular characterization and risk analysis of *Giardia duodenalis* assemblages in corticosteroid-treated and non-treated patients in Ismailia, Arab Republic of Egypt

**DOI:** 10.1186/s13099-025-00680-w

**Published:** 2025-03-11

**Authors:** Shahira Abdelaziz Ali Ahmed, Amira Bakr Mokhtar, Samar Farag Mohamed, Marwa Ibrahim Saad El‑Din, Catherine O.’Dowd Phanis, Stefani Kazamia, Chad Schou, Paweł Gładysz, Anna Lass, Annalisa Quattrocchi, Panagiotis Karanis, Samer Eid Mohamed Gad

**Affiliations:** 1 Department of Parasitology, Faculty of Medicine, Suez Canal University, Ismailia, 41522 Egypt; 2 Department of Family Medicine, Faculty of Medicine, Suez Canal University, Ismailia, 41522 Egypt; 3 Invertebrates- Zoology Department, Faculty of Science, Suez Canal University, Ismailia, 41522 Egypt; 4 Department of Basic and Clinical Sciences, University of Nicosia Medical School, 24005, CY-1700 Nicosia, Cyprus; 5 Department of Tropical Medicine and Parasitology, Institute of Maritime and Tropical Medicine in Gdynia, Gdynia, Poland; 6 Department of Primary Care and Population Health, University of Nicosia Medical School, 24005, CY-1700 Nicosia, Cyprus; 7 Medical Faculty and University Hospital, University of Cologne, Cologne, Germany

**Correction: Gut Pathogens (2024) 16:74** 10.1186/s13099-024-00668-y

In this article [1], inadvertently, dashes were converted to beta symbol. The incorrect and corrected Table 4 are given below.

Incorrect Table 4:

**Table 4:** *Giardia duodenalis* assemblages detected in patients on corticosteroid therapy (POCT) and controls (CONT)

No.	Group	Serial	Symp. Assoc.	*tpi*	*bg*	*gdh*	Assemblage detected	positive/total
1	POCT	A21	1	ββ	β	B	B	15/21
2	POCT	A22	1	β	β	β	β	
3	POCT	A23	2	β	β	β	β	
4	POCT	A24	2	β	β	B	B	
5	POCT	A25	2	B	β	β	B	
6	POCT	A26	2	β	β	β	β	
7	POCT	A27	2	β	β	B	B	
8	POCT	A28	1	A	β	β	A	
9	POCT	A29	2	A	β	β	A	
10	POCT	A30	1	β	β	AII	A	
11	POCT	A31	1	β	β	β	β	
12	POCT	A32	2	A	β	B	A + B	
13	POCT	A33	2	A	β	β	A	
14	POCT	A34	2	A	β	B	A + B	
15	POCT	A35	2	A^a^	ββ	β	A	
16	POCT	A36	2	ββ	β	AII	A	
17	POCT	A37	1	β	β	β	β	
18	POCT	A38	1	B	β	β	B	
19	POCT	A39	1	A	β	B	A + B	
20	POCT	A40	1	β	β	β	β	
21	POCT	A41	2	A	β	ββ	A	
22	CONT	B01	1	β	B	AII	A + B	23/31
23	CONT	B02	1	β	β	β	β	
24	CONT	B03	1	B	B	B	B	
25	CONT	B04	1	β	β	β	β	
26	CONT	B05	1	β	β	β	β	
27	CONT	B06	1	A	β	β	A	
28	CONT	B07	1	β	β	β	β	
29	CONT	B08	1	β	β	β	β	
30	CONT	B09	1	β	β	β	β	
31	CONT	B10	1	β	β	β	β	
32	CONT	B11	1	β	β	β	β	
33	CONT	C12	2	A	AII	AII	A	
34	CONT	C13	2	B	B	B	B	
35	CONT	C14	2	B	B	AII	A + B	
36	CONT	C15	2	A	B	β	A + B	
37	CONT	C16	2	B	AII	ββ	A + B	
38	CONT	C17	2	B	B	E	B + E	
39	CONT	C18	2	B	B	β	B	
40	CONT	C19	2	B	B	β	B	
41	CONT	C20	2	B	B	B	B	
42	CONT	D42	1	β	B	E	B + E	
43	CONT	D43	1	–	B	–	B	
44	CONT	D44	1	A	AII	–	A	
45	CONT	D45	2	–	B	–	B	
46	CONT	D46	2	B	B^a^	B	B	
47	CONT	D47	2	B	B^a^	AII	A + B	
48	CONT	D48	2	B	B	B	B	
49	CONT	D49	2	B	B	B	B	
50	CONT	D50	1	B	B	B	B	
51	CONT	D51	1	B	B^a^	E	B + E	
52	CONT	D52	2	A	B	–	A + B	
Total				29/52	22/52	22/52	38/52	

*POCT* Patients on corticosteroid therapy, *CONT* Controls, *Symp. Assoc.* Symptoms association; 1: Symptomatic; 2: Asymptomatic

^a^Sequence excluded from the alignment due to poor coverage; –: not amplified.

Correct Table 4:

**Table 4:** *Giardia duodenalis* assemblages detected in patients on corticosteroid therapy (POCT) and controls (CONT)

No.	Group	Serial	Symp. Assoc.	*tpi*	*bg*	*gdh*	Assemblage detected	Positive/total
1	POCT	A21	1	–	–	B	B	15/21
2	POCT	A22	1	–	–	–	–	
3	POCT	A23	2	–	–	–	–	
4	POCT	A24	2	–	–	B	B	
5	POCT	A25	2	B	–	–	B	
6	POCT	A26	2	–	–	–	–	
7	POCT	A27	2	–	–	B	B	
8	POCT	A28	1	A	–	–	A	
9	POCT	A29	2	A	–	–	A	
10	POCT	A30	1	–	–	AII	A	
11	POCT	A31	1	–	–	–	–	
12	POCT	A32	2	A	–	B	A + B	
13	POCT	A33	2	A	–	–	A	
14	POCT	A34	2	A	–	B	A + B	
15	POCT	A35	2	A^a^	–	–	A	
16	POCT	A36	2	–	–	AII	A	
17	POCT	A37	1	–	–	–	–	
18	POCT	A38	1	B	–	–	B	
19	POCT	A39	1	A	–	B	A + B	
20	POCT	A40	1	–	–	–	–	
21	POCT	A41	2	A	–	–	A	
22	CONT	B01	1	–	B	AII	A + B	23/31
23	CONT	B02	1	–	–	–	–	
24	CONT	B03	1	B	B	B	B	
25	CONT	B04	1	–	–	–	–	
26	CONT	B05	1	–	–	–	–	
27	CONT	B06	1	A	–	–	A	
28	CONT	B07	1	–	–	–	–	
29	CONT	B08	1	–	–	–	–	
30	CONT	B09	1	–	–	–	–	
31	CONT	B10	1	–	–	–	–	
32	CONT	B11	1	–	–	–	–	
33	CONT	C12	2	A	AII	AII	A	
34	CONT	C13	2	B	B	B	B	
35	CONT	C14	2	B	B	AII	A + B	
36	CONT	C15	2	A	B	–	A + B	
37	CONT	C16	2	B	AII	–	A + B	
38	CONT	C17	2	B	B	E	B + E	
39	CONT	C18	2	B	B	–	B	
40	CONT	C19	2	B	B	–	B	
41	CONT	C20	2	B	B	B	B	
42	CONT	D42	1	–	B	E	B + E	
43	CONT	D43	1	–	B	–	B	
44	CONT	D44	1	A	AII	–	A	
45	CONT	D45	2	–	B	–	B	
46	CONT	D46	2	B	B^a^	B	B	
47	CONT	D47	2	B	B^a^	AII	A + B	
48	CONT	D48	2	B	B	B	B	
49	CONT	D49	2	B	B	B	B	
50	CONT	D50	1	B	B	B	B	
51	CONT	D51	1	B	B^a^	E	B + E	
52	CONT	D52	2	A	B	–	A + B	
Total				29/52	22/52	22/52	38/52	

^a^Sequence excluded from the alignment due to poor coverage; –: Not amplified

*POCT* Patients on corticosteroid therapy, *CONT* Controls, *Symp. Assoc*. Symptoms association, *1* Symptomatic, *2* Asymptomatic

The original article has been corrected.
