# Supplementary material for: Molecular characterization and risk analysis of Giardia duodenalis assemblages in corticosteroid-treated and non-treated patients in Ismailia, Arab Republic of Egypt
Source: Gut Pathog. 2024 Dec 13;16:74. doi: 10.1186/s13099-024-00668-y (PMC11645789; doi:10.1186/s13099-024-00668-y)
Supplement: Supplementary file 1 — Additional file 1 [file 13099_2024_668_MOESM1_ESM.docx]

**Supplementary File.**

**Table S1:** Anamnesis of patients on corticosteroid therapy (POCT).

| No. | ID | Gender/age | Disease | RoA | Type of CST | Complication |
| --- | --- | --- | --- | --- | --- | --- |
| P1 | A21 | F/65 | RA | Local & oral | Prednisolone | Weight gain, rash |
| P2 | A28 | F/21 | BA | Oral & inhalation | NI | None |
| P3 | A25 | F/67 | RA  BA | Oral & inhalation | NI | None |
| P4 | A26 | M/10 | AR | Oral | Dexamethasone | None |
| P5 | A27 | F/6 | ND | Oral | Prednisolone | Weight gain |
| P6 | A24 | F/70 | RA | Local & oral | Prednisolone | None |
| P7 | A29 | M/40 | IBD | Oral | Prednisolone | Peptic ulcer |
| P8 | A30 | F/10 | BA | Inhalation | NI | None |
| P9 | A31 | F/62 | RA | Local & oral | Prednisolone | Weight gain, peptic ulcer, & osteoporosis |
| P10 | A32 | M/3 | AR | Local & oral | NI | None |
| P11 | A33 | F/10 | AR | Oral | Others | None |
| P12 | A34 | F/10 | BA | Inhalation | NI | None |
| P13 | A35 | F/6 | AR | Oral | NI | None |
| P14 | A36 | M/2 | AR | Oral | NI | None |
| P15 | A23 | F/9 | AR | Local & oral | Others | None |
| P16 | A38 | M/7 | BA | Inhalation | NI | None |
| P17 | A39 | F/10 | CC | Oral | Others | None |
| P18 | A40 | M/8 | AR | Oral | Others | None |
| P19 | A41 | M/10 | ND | Oral | Others | Weight gain |
| P20 | A37 | M/10 | BA | Inhalation | NI | None |
| P21 | A22 | F/20 | AR | Local & oral | NI | None |

F: Female; M: Male; RoA: Route of administration; NI: Not identified; BA: Biliary atresia; RA: Rheumatoid arthritis; ND: Nephrotic disease; AR: Allergic rhinitis; CC: Chronic cough; CST: Corticosteroid; IBD: Inflammatory bowel disease.


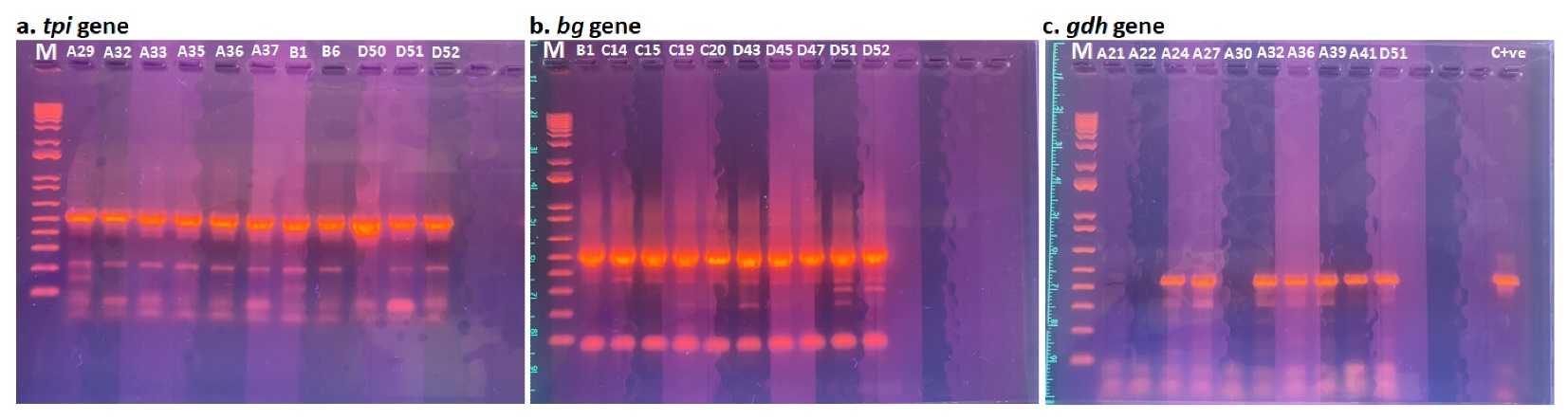


**Figure S1:** Nested PCR amplification of *tpi*, *bg* and *gdh* genes from a subset of the *G. duodenalis* isolates. M: 100 bp DNA ladder; Lanes' alphabetical enumerations correspond to the ID of the isolate; C+ve: Positive control.

**Table S2:** *Giardia* infection assemblages by patients’ characteristics (univariate analysis).

| **Variable** | **Categories** | **Assemblage A (N=10)** | | **Assemblage B (N=16)** | | **Assemblage Mixed (N=12)** | | **p-trend** |
| --- | --- | --- | --- | --- | --- | --- | --- | --- |
|  |  | N | % | N | % | N | % |  |
| Sex | Male | 6 | 60.0 | 10 | 62.5 | 7 | 58.3 | 0.975 |
|  | Female | 4 | 40.0 | 6 | 37.5 | 5 | 41.7 |  |
| Mean Age (SD) |  | 11.4 (11.4) | | 17.5 (24.8) | | 6.7 (2.6) | | 0.271 |
| Residence | Urban | 0 | 0.0 | 5 | 31.3 | 5 | 41.7 | 0.061 |
|  | Rural | 10 | 100.0 | 11 | 68.8 | 7 | 58.3 |  |
| Symptomatic | Yes | 4 | 40.0 | 5 | 31.3 | 5 | 41.7 | 0.835 |
|  | No | 6 | 60.0 | 11 | 68.8 | 7 | 58.3 |  |
| Abdominal pain | Yes | 4 | 100.0 | 4 | 80.0 | 2 | 40.0 | 0.231 |
|  | No | 0 | 0.0 | 1 | 20.0 | 3 | 60.0 |  |
| Diarrhea | Yes | 2 | 50.0 | 0 | 0.0 | 3 | 60.0 | 0.151 |
|  | No | 2 | 50.0 | 5 | 100.0 | 2 | 40.0 |  |
| Other symptoms* | Yes | 2 | 50.0 | 2 | 40.0 | 3 | 60.0 | 1.000 |
|  | No | 2 | 50.0 | 3 | 60.0 | 2 | 40.0 |  |
| Have domestic animals | Yes | 5 | 50.0 | 8 | 50.0 | 3 | 25.0 | 0.354 |
|  | No | 5 | 50.0 | 8 | 50.0 | 9 | 75.0 |  |
| Have water supply | Yes | 8 | 80.0 | 14 | 87.5 | 11 | 91.7 | 0.708 |
|  | No | 2 | 20.0 | 2 | 12.5 | 1 | 8.3 |  |
| Have sewage system | Yes | 7 | 70.0 | 12 | 75.0 | 9 | 75.0 | 1.000 |
|  | No | 3 | 30.0 | 4 | 25.0 | 3 | 25.0 |  |
| Concomitant parasitic infection | Mixed | 5 | 50.0 | 5 | 31.3 | 3 | 25.0 | 0.472 |
|  | Single | 5 | 50.0 | 11 | 68.8 | 9 | 75.0 |  |

*Other symptoms refer to epigastric pain, postprandial heart burn, constipation, loss of weight, nausea, pallor; SD: Standard deviation.

**Table S3:** Assemblages identified based on BLAST search and phylogenetic assay for triosephosphate isomerase (*tpi*) partial coding sequences.

| **Group** | **ID** | **Accession** | **Length** | **(Sub)assemblage by Phylogeny** | **Assemblage by BLAST Analysis** | **Top Match Accession** | **Identity [%]** | **Query Cover [%]** | **E-value** |
| --- | --- | --- | --- | --- | --- | --- | --- | --- | --- |
| POCT | A25 | PP566746 | 379 | B | B | LC507535.1 | 100.00 | 100.00 | 0.0 |
| POCT | A28 | PP566747 | 426 | A | A | MN844148.1 | 100.00 | 100.00 | 0.0 |
| POCT | A29 | PP566748 | 404 | A | A | MN844148.1 | 99.75 | 100.00 | 0.0 |
| POCT | A32 | PP566749 | 403 | A | A | MN844148.1 | 100.00 | 100.00 | 0.0 |
| POCT | A33 | PP566750 | 404 | A | A | MN844148.1 | 100.00 | 100.00 | 0.0 |
| POCT | A34 | PP566751 | 454 | A | A | MN844148.1 | 99.34 | 100.00 | 0.0 |
| POCT | A35 | PP566752 | 334 | A | A | MN844148.1 | 100.00 | 100.00 | 5.00E-172 |
| POCT | A38 | PP566753 | 454 | B | B | MG736282.1 | 100.00 | 100.00 | 0.0 |
| POCT | A39 | PP566754 | 426 | A | A | MN844148.1 | 99.77 | 100.00 | 0.0 |
| POCT | A41 | PP566755 | 327 | A | A | MN844148.1 | 99.69 | 100.00 | 2.00E-166 |
| CONT | B03 | PP566756 | 351 | B | B | OR921177.1 | 98.86 | 100.00 | 5.00E-177 |
| CONT | B06 | PP566757 | 430 | A | A | MN844148.1 | 100.00 | 100.00 | 0 |
| CONT | C12 | PP566758 | 397 | A | A | MN844148.1 | 100.00 | 100.00 | 0 |
| CONT | C13 | PP566759 | 378 | B | B | MG924459.1 | 99.47 | 100.00 | 0 |
| CONT | C14 | PP566760 | 351 | B | B | LC507535.1 | 100.00 | 100.00 | 0 |
| CONT | C15 | PP566761 | 325 | A | A | MN844148.1 | 100.00 | 100.00 | 5.00E-167 |
| CONT | C16 | PP566762 | 396 | B | B | MG924459.1 | 100.00 | 100.00 | 0 |
| CONT | C17 | PP566763 | 368 | B | B | LC507535.1 | 99.73 | 100.00 | 0 |
| CONT | C18 | PP566764 | 351 | B | B | LC507535.1 | 99.15 | 100.00 | 4.00E-178 |
| CONT | C19 | PP566765 | 454 | B | B | MG924459.1 | 99.78 | 100.00 | 0 |
| CONT | C20 | PP566766 | 454 | B | B | OQ968712.1 | 99.78 | 100.00 | 0 |
| CONT | D44 | PP566767 | 454 | A | A | MN844148.1 | 99.78 | 100.00 | 0 |
| CONT | D46 | PP566768 | 454 | B | B | MG924459.1 | 100.00 | 100.00 | 0 |
| CONT | D47 | PP566769 | 460 | B | B | MG736282.1 | 99.57 | 100.00 | 0 |
| CONT | D48 | PP566770 | 460 | B | B | MK962818.1 | 99.57 | 100.00 | 0 |
| CONT | D49 | PP566771 | 369 | B | B | LC507535.1 | 99.73 | 100.00 | 0 |
| CONT | D50 | PP566772 | 285 | B | B | PP388936.1 | 99.65 | 100.00 | 9.00E-144 |
| CONT | D51 | PP566773 | 369 | B | B | MG924459.1 | 100.00 | 100.00 | 0 |
| CONT | D52 | PP566774 | 404 | A | A | MN844148.1 | 99.75 | 100.00 | 0 |

POCT: Patients on corticosteroid therapy; CONT: Control individuals.

**Table S4:** Assemblages identified based on BLAST search and phylogenetic assay for beta-giardin (*bg*) partial coding sequences.

| **Group** | **ID** | **Accession** | **Length** | **(Sub)assemblage by Phylogeny** | **Assemblage by BLAST Analysis** | **Top Match Accession** | **Identity [%]** | **Query Cover [%]** | **E-value** |
| --- | --- | --- | --- | --- | --- | --- | --- | --- | --- |
| CONT | B01 | PP566775 | 383 | B | B | FJ971460.1 | 99.74 | 100 | 0 |
| CONT | B03 | PP566776 | 404 | B | B | FJ971460.1 | 99.75 | 100 | 0 |
| CONT | C12 | PP566777 | 362 | AII | A | MG924451.1 | 99.45 | 100 | 0 |
| CONT | C13 | PP566778 | 373 | B | B | MG924453.1 | 99.46 | 100 | 0 |
| CONT | C14 | PP566779 | 459 | B | B | FJ971458.1 | 99.34 | 99 | 0 |
| CONT | C15 | PP566780 | 345 | B | B | MG924453.1 | 99.42 | 100 | 6.00E-176 |
| CONT | C16 | PP566781 | 435 | A | A | MG924452.1 | 100.00 | 100 | 0 |
| CONT | C17 | PP566782 | 451 | B | B | FJ971458.1 | 100.00 | 100 | 0 |
| CONT | C18 | PP566783 | 417 | B | B | FJ971460.1 | 100.00 | 100 | 0 |
| CONT | C19 | PP566784 | 383 | B | B | FJ971460.1 | 99.74 | 100 | 0 |
| CONT | C20 | PP566785 | 385 | B | B | FJ971460.1 | 99.48 | 100 | 0 |
| CONT | D42 | PP566786 | 462 | B | B | FJ971458.1 | 100.00 | 100 | 0 |
| CONT | D43 | PP566787 | 403 | B | B | FJ971460.1 | 99.50 | 100 | 0 |
| CONT | D44 | PP566788 | 434 | AII | A | MG924451.1 | 99.77 | 100 | 0 |
| CONT | D45 | PP566789 | 393 | B | B | FJ971460.1 | 99.75 | 100 | 0 |
| CONT | D46 | PP566790 | 285 | B | B | MG736249.1 | 98.95 | 100 | 2.00E-140 |
| CONT | D47 | PP566791 | 203 | B | B | MN174847.1 | 100.00 | 100 | 2.00E-99 |
| CONT | D48 | PP566792 | 429 | B | B | KT948086.1 | 99.77 | 100 | 0 |
| CONT | D49 | PP566793 | 331 | B | B | MG924453.1 | 99.70 | 100 | 3.00E-169 |
| CONT | D50 | PP566794 | 433 | B | B | FJ971458.1 | 100.00 | 100 | 0 |
| CONT | D51 | PP566795 | 204 | B | B | MG924453.1 | 99.51 | 100 | 6.00E-99 |
| CONT | D52 | PP566796 | 366 | B | B | FJ971460.1 | 99.73 | 100 | 0 |

CONT: Control individuals.

**Table S5:** Assemblages identified based on BLAST search and phylogenetic assay for glutamate dehydrogenase (*gdh*) partial coding sequences.

| **Group** | **ID** | **Accession** | **Length** | **(Sub)assemblage by Phylogeny** | **Assemblage by BLAST Analysis** | **Top Match Accession** | **Identity [%]** | **Query Cover [%]** | **E-value** |
| --- | --- | --- | --- | --- | --- | --- | --- | --- | --- |
| POCT | A21 | PP576000 | 308 | B | B | LC507350.1 | 98.05 | 100 | 8.00E-150 |
| POCT | A24 | PP576001 | 368 | B | B | MH765413.1 | 99.73 | 100 | 0 |
| POCT | A27 | PP576002 | 368 | B | B | KC313930.1 | 99.73 | 99 | 0 |
| POCT | A30 | PP576003 | 299 | AII | A | MN996368.1 | 99.33 | 100 | 3.00E-149 |
| POCT | A32 | PP576004 | 375 | B | B | KC313930.1 | 99.73 | 99 | 0 |
| POCT | A34 | PP576005 | 308 | B | B | LC507350.1 | 99.68 | 100 | 2.00E-156 |
| POCT | A36 | PP576006 | 375 | AII | A | OQ446153.1 | 99.47 | 99 | 0 |
| POCT | A39 | PP576007 | 375 | B | B | KC313930.1 | 99.46 | 99 | 0 |
| CONT | B01 | PP576008 | 269 | AII | A | MN996368.1 | 100.00 | 100 | 5.00E-136 |
| CONT | B03 | PP576009 | 295 | B | B | LC507350.1 | 98.98 | 100 | 2.00E-146 |
| CONT | C12 | PP576010 | 297 | AII | A | MN996368.1 | 100.00 | 100 | 2.00E-151 |
| CONT | C13 | PP576011 | 295 | B | B | LC507350.1 | 99.66 | 100 | 9.00E-149 |
| CONT | C14 | PP576012 | 372 | AII | A | MN996368.1 | 99.73 | 100 | 0 |
| CONT | C17 | PP576013 | 370 | E | E | MK561343.1 | 99.46 | 100 | 0 |
| CONT | C20 | PP576014 | 368 | B | B | MH765408.1 | 100.00 | 100 | 0 |
| CONT | D42 | PP576015 | 370 | E | E | MK561343.1 | 99.19 | 100 | 0 |
| CONT | D46 | PP576016 | 367 | B | B | KC313930.1 | 99.45 | 99 | 0 |
| CONT | D47 | PP576017 | 307 | AII | A | MN996368.1 | 99.67 | 100 | 6.00E-156 |
| CONT | D48 | PP576018 | 267 | B | B | LC507392.1 | 98.88 | 100 | 5.00E-131 |
| CONT | D49 | PP576019 | 321 | B | B | LC507346.1 | 99.69 | 100 | 1.00E-163 |
| CONT | D50 | PP576020 | 370 | B | B | HM136880.1 | 99.46 | 99 | 0 |
| CONT | D51 | PP576021 | 373 | E | E | MK561343.1 | 99.20 | 100 | 0 |

POCT: Patients on corticosteroid therapy; CONT: Control individuals.

**Table S6:** *Giardia duodenalis* genotyping data in Arab Republic of Egypt (ARE) population.

| **Governorate** | **Type of study** | **Target population** | **Method of analysis** | **Target gene (s)** | **Assem. detected** | **Sub-assem.** | **Prevalent assem.** | **Intra-assem.**  **heterogeneity** | **Reference** |
| --- | --- | --- | --- | --- | --- | --- | --- | --- | --- |
| Ismailia | Case control study | POCT  CONT | Microscopy,  PCR &  Sequencing | *tpi*, *bg*  *gdh* | A  B  A+B^*^  B+E^*^ | AII | A in POCT  B in CONT | N/I | **The present study** |
| Ismailia | Cross sectional | Diarrhoea children | ICT,  PCR &  Sequencing | *tpi*, *bg*  *gdh* | A  B  A+B  A+E | AI  AII | B | B with high variability | ]1[ |
| Ismailia | Cross sectional | GIT symptomatic children | Microscopy,  PCR &  Sequencing | *tpi*, *bg*  *gdh* | A  B  C  A+B | AI/AII  BIII/BIV | B | B with high variability | ]2[ |
| Ismailia | Case control | Pre-school children with IDA  Pre-school Children non-anaemic | Microscopy, IGS-PCR & HRMC | *IGS* | A  B  A+B | AI  AII | A | N/I | ]3[ |
| Kafr El-Sheikh | Cross sectional | Outpatients | Microscopy,  PCR &  Sequencing | *tpi*, *bg*  *gdh* | A  B  A+B | AII  BIII | B | B with high variability | ]4[ |
| Al-Amereyah, Gharbia & Kafr El Sheik | Cross sectional | NA | Microscopy,  PCR &  Sequencing | *tpi* | A  B  E  B+E | N/A | B | N/A | ]5[ |
| Fayoum | Cross sectional | GIT symptomatic children | Microscopy,  Assem. E-specific PCR &  Sequencing | *tpi* | E | N/A | E | N/A | ]6[ |
| El-Dakahlia, El-Gharbia, & Damietta | Cross sectional | Children in childcare centres | PCR &  Sequencing | *tpi*, *bg*  *gdh* | A  B  A+B | AII, AIII | B | B with high variability | ]7[ |
| El-Sharkia | Cross sectional | GIT symptomatic children | Microscopy,  PCR &  Sequencing | *tpi* | A  B | N/A | B | N/A | ]8[ |
| Mansoura | Case control study | Symptomatic were divided into:  Giardia +ve children (NITR & ITR)  Giardia -ve children (NITR & ITR) | Microscopy &  PCR | *tpi* | A  B  A+B | N/I | A | N/I | ]9[ |
| Kafr El Sheikh | Cross sectional | Outpatient | Microscopy,  PCR &  Sequencing | *tpi* | A  B  E | AI, AII | B | A, B, E with high variability | ]10[ |
| El-Sharkia | Cross sectional | GIT symptomatic children | Microscopy,  PCR &  Sequencing | *tpi* | A  B | AII  BIII, BIV | B | N/A | ]11[ |
| Cairo | Cross sectional | Children | Microscopy,  PCR &  Sequencing | *tpi* | A  B | N/A | B |  | ]12[ |
| Upper Egypt | Cross sectional | Children | Assem. A, B & E-specific PCR | *tpi* | A  B  A+B | N/I | A | N/I | ]13[ |
| West Delta region | Cross sectional | Children | Microscopy, Assem. A & B-specific PCR | *tpi* | A  B  A+B | N/I | B | N/I | ]14[ |
| Beni-Suef Governorate | Cross sectional | Children with diarrhea | Microscopy,  PCR &  Sequencing | *tpi* | A  B | AII  BIII, BIV | B | B with high variability | ]15[ |
| Cairo | Cross sectional | Children with diarrhea | Microscopy,  Real time PCR, PCR &  Sequencing | *tpi*, *bg*  *gdh* | A  B  A+B | AII | B | N/I | ]16[ |
| Dakahalia Governorate | Cross sectional | Children with diarrhea | Microscopy,  PCR, PCR-RFLP | *tpi* | A  B  A+B | N/I | B | N/I | ]17[ |
| Kafr El-Sheikh | Cross sectional | Outpatient | Microscopy,  PCR, real time PCR | *tpi*, *bg*  *gdh* | A  B | AII  BIII | A | N/I | ]18[ |

POCT: Patients on corticosteroid therapy; CONT: Control individuals; IDA: Iron deficiency anemia; ^*^ Mixed infection based on different loci; ICT: Immunochromatography; PCR: Polymerase chain reaction; *tpi*: triosephosphate isomerase; *bg*: beta-giardin; *gdh*: glutamate dehydrogenase; Assem.: Assemblage; N/A: Data not available; N/I: Not investigated; ITR: Immunosuppressive therapy recipients; NITR: Non-immunosuppressive therapy recipients.

**References:**

1. Helmy YA, Klotz C, Wilking H, Krücken J, Nöckler K, Von Samson-Himmelstjerna G, Zessin KH, Aebischer T. Epidemiology of *Giardia duodenalis* infection in ruminant livestock and children in the Ismailia province of Egypt: Insights by genetic characterization. Parasit Vectors. 2014; 7: doi: 10.1186/1756-3305-7-321

2. Soliman RH, Fuentes I, Rubio JM. Identification of a novel assemblage B subgenotype and a zoonotic assemblage C in human isolates of *Giardia intestinalis* in Egypt. Parasitol Int. 2011; 60:507–511. doi: 10.1016/j.parint.2011.09.006

3. Hussein EM, Ismail OA, Mokhtar AB, Mohamed SE, Saad RM. Nested PCR targeting intergenic spacer (IGS) in genotyping of *Giardia duodenalis* isolated from symptomatic and asymptomatic infected Egyptian school children. Parasitol Res. 2017; 116:763–771. doi: 10.1007/S00436-016-5347-0

4. Yu F, Amer S, Qi M, Wang R, Wang Y, Zhang S, Jian F, Ning C, El Batae H, Zhang L. Multilocus genotyping of *Giardia duodenalis* isolated from patients in Egypt. Acta Trop. 2019; 196:66–71. doi: 10.1016/j.actatropica.2019.05.012

5. Foronda P, Bargues MD, Abreu-Acosta N, Periago M V., Valero MA, Valladares B, Mas-Coma S. Identification of genotypes of *Giardia intestinalis* of human isolates in Egypt. Parasitol Res. 2008; 103:1177–1181. doi: 10.1007/s00436-008-1113-2

6. Abdel-Moein KA, Saeed H. The zoonotic potential of *Giardia intestinalis* assemblage E in rural settings. Parasitol Res. 2016; 115:3197–3202. doi: 10.1007/s00436-016-5081-7

7. Naguib D, El-Gohary AH, Roellig D, Mohamed AA, Arafat N, Wang Y, Feng Y, Xiao L. Molecular characterization of *Cryptosporidium* spp. and *Giardia duodenalis* in children in Egypt. Parasit Vectors. 2018; 11: doi: 10.1186/s13071-018-2981-7

8. Mohamed AMA, Bayoumy AM, Abo-hashim AH, Ibrahim AA, El-Badry AA. Giardiasis in symptomatic children from Sharkia, Egypt: genetic assemblages and associated risk factors. Journal of Parasitic Diseases. 2020; 44:719–724. doi: 10.1007/s12639-020-01254-0

9. Ismail A, Abdel-Magied AA, Elhenawy AA, El-Nahas HA. Association between *Giardia* genotype and oxidative stress biomarkers among *Giardia*-infected children: A case–control study. Acta Parasitol. 2022; 67:1145–1151. doi: 10.1007/s11686-022-00548-y

10. Amer SER. 33-146 Genotypic and phylogenetic characterization of *Giardia intestinalis* from human and dairy cattle in Kafr El Sheikh Governorate, Egypt. 2013. 2013 p.

11. El-Badry A, Mohammed F, Abdul Gawad E. Predominance of *Giardia intestinalis* assemblage B in diarrhoeic children in Sharkia, Egypt. Parasitologists United Journal. 2017; 10:39–43. doi: 10.21608/puj.2017.4735

12. Taha SA, Abd Z, Aal AL, Saleh NS, El-Badry AA. *Giardia intestinalis* assemblages among Egyptian symptomatic children: Prevalence and seasonal distribution in Cairo, Egypt. 2018; 661–668 p.

13. Ahmad AA, El-Kady AM, Hassan TM. Genotyping of *Giardia duodenalis* in children in upper Egypt using assemblage- specific PCR technique. PLoS One. 2020; 15:e0240119. doi: 10.1371/JOURNAL.PONE.0240119

14. Elhadad H, Abdo S, Salem A, Mohamed M, El-Taweel H, El-Abd E. Comparison of gdh polymerase chain reaction-restriction fragment length polymorphism and tpi assemblage-specific primers for characterization of *Giardia intestinalis* in children. Trop Parasitol. 2022; 12:41–47. doi: 10.4103/TP.TP_28_21

15. Ghieth MA, El-Badry AA, Abu-Sarea EY, Abdel Gawad SS, Elsharkawy MM. Genotypic analysis of *Giardia duodenalis* in children at Egypt. Comp Clin Path. 2016; 25:1241–1246. doi: 10.1007/S00580-016-2337-7/METRICS

16. Fahmy HM, El-Serougi AO, El Deeb HK, Hussein HM, Abou-Seri HM, Klotz C, Aebischer T, El Sayed Khalifa Mohamed K. *Giardia duodenalis* assemblages in Egyptian children with diarrhea. European Journal of Clinical Microbiology and Infectious Diseases. 2015; 34:1573–1581. doi: 10.1007/s10096-015-2389-7

17. El-Tantawy N, Taman A. The epidemiology of *Giardia intestinalis* assemblages A and B among Egyptian children with diarrhea: A PCR-RFLP-based approach. Parasitologists United Journal. 2014; 7:104. doi: 10.4103/1687-7942.149557

18. Elmahallawy EK, Gareh A, Ghallab MMI, Köster PC, Dashti A, Aboelsoued D, Toaleb NI, Alzaylaee H, Gonzálvez M, Saleh AA, et al. Microscopy detection and molecular characterisation of *Giardia duodenalis* infection in outpatients seeking medical care in Egypt. Front Public Health. 2024; 12: doi: 10.3389/fpubh.2024.1377123
